# *EGFR* mutation testing in blood for guiding EGFR tyrosine kinase inhibitor treatment in patients with nonsmall cell lung cancer: A protocol for systematic review and meta-analysis

**DOI:** 10.1097/MD.0000000000006151

**Published:** 2017-02-17

**Authors:** Jin-Qiu Yuan, Yue-Lun Zhang, Hai-Tao Li, Chen Mao

**Affiliations:** a School of Public Health and Primary Care, Faculty of Medicine, The Chinese University of Hong Kong, Hong Kong; b School of Medicine, Shenzhen University, Shenzhen, China.

**Keywords:** *EGFR* mutation, meta-analysis, nonsmall cell lung cancer, plasma, serum, systematic review

## Abstract

**Background:**

Epidermal growth factor receptor (*EGFR*) mutation testing in tumor tissue is now a common practice in selecting non-small cell lung cancer (NSCLC) patients for EGFR tyrosine kinase inhibitor (TKI) treatment. However, tumor tissues are often absent or insufficient for the testing. Blood is a potential substitute providing a noninvasive, easily accessible and repeatedly measureable source of genotypic information. However which is the best blood EGFR mutation testing method remains unclear. We undertake this study to investigate the best blood EGFR mutation testing method for selecting EGFR TKI treatment in patients with NSCLC.

**Methods:**

This study was registered in PROSPERO (CRD42017055263). PubMed, EMBASE, Cochrane library, and NIHR Health Technology Assessment program will be searched. Studies fulfill the following criteria will be eligible: (1) randomized controlled trials or cohort studies; (2) included patients with NSCLC; (3) reported response, progression-free survival, or overall survival for EGFR TKI by the *EGFR* mutation status in blood sample. Diagnostic accuracy of blood *EGFR* mutation tests for predicting response to TKI will be pooled. Tumor response, progression-free survival, and overall survival according to different blood *EGFR* mutation testing methods will be evaluated and compared.

**Results:**

Based published data and combined analysis, this study will quantitatively compare the blood *EGFR* mutation testing methods according to their accuracy for predicting treatment response and relationship with clinical outcome in NSCLC patients treated with EGFR TKIs.

**Conclusion:**

This protocol will determine the best blood *EGFR* mutation testing method.

## Abstract 2

Supplemental Digital Content is available in the text

## 1 Introduction

Nonsmall cell lung cancer (NSCLC) is one of the most common cancers and the 1st leading cause of cancer-related mortality in the world.^[1]^ To date 2 tyrosine kinase inhibitors (TKIs) targeting epidermal growth factor receptor (*EGFR*), gefitinib and erlotinib, have been approved for patients with locally advanced or metastatic NSCLC.^[2]^ However, only about 10% of NSCLC patients demonstrate clinically relevant benefits from EGFR-TKI treatment. Since 2004, several research groups have reported an association between mutations in the EGFR gene's kinase domain and EGFR TKI sensitivity, indicating that TKIs are especially effective in patients with activating *EGFR* mutations.^[3,4]^*EGFR* mutations have been widely used as a biomarker to select patients for EGFR TKI treatment.

*EGRF* mutations status is commonly tested in tumor tissues. However, it is often difficult to obtain sufficient tumor tissues for *EGFR* mutation analyses from patients with advanced NSCLC who are not candidates for surgery. Lacking of tissue sample is a significant limitation, even in prospectively conducted clinical trials, less than 50% of the patients had sufficient tumor tissues available for *EGFR* mutations analyses.^[5]^ Interest has been stimulated in *EGFR* mutations analyses using surrogate samples such as blood. Several research groups have detected *EGFR* mutations in plasma DNA^[6,7]^ or serum DNA^[8,9]^ and found a high correlation between *EGFR* mutations status in plasma or serum and tumor tissue. Accumulating evidence has also indicated that *EGFR* mutations in blood could potentially predict treatment response and survival.^[6,8,9]^

Currently, a number of methods are available for testing *EGFR* mutations in blood samples, including direct sequencing, amplification refractory mutation system, denaturing high performance liquid chromatography, mutant-enriched polymerase chain reaction, high resolution melt, mutant-enriched liquidchip, and Allele-Specific Arrayed Primer Extension. As a variety of methods are now available for testing *EGFR* mutations in blood, interest has been growing in investigating the most appropriate *EGFR* mutation testing method. One study comparing 3 different methods for analyzing *EGFR* mutations in blood samples suggested that *EGFR* mutations detected by Scorpion-amplification refractory mutation system in blood were better predictors of response rate to EGFR TKI than mutations detected with denaturing high performance liquid chromatography and mutant-enriched liquidchip.^[10]^ Another study comparing SARMS and WAVE/Surveyor methods in detecting *EGFR* mutations in plasma showed very low concordance between the 2 methods.^[11]^ These studies suggested that different *EGFR* mutation testing methods may have significantly different clinical value in selecting appropriate patients to receive EGFR TKI treatment. However, there is still insufficient evidence evaluating the clinical outcomes of EGFR TKI treatment according to mutations identified through different blood testing methods.

The aims of this systemic review are to evaluate and compare the accuracy of different blood *EGFR* mutation testing methods for predicting response to EGFR TKI; to assess the clinical outcomes of EGFR TKI treatment according to blood *EGFR* mutation testing methods.

## 2 Methods

This study is a systematic review and meta-analysis. This protocol was performed according to Preferred Reporting Items for Systematic review and Meta-Analysis Protocols (PRISMAP).^[12]^ The study was registered in PROSPERO International prospective register of systematic reviews (CRD42017055263).^[13]^ Because this is a literature-based study, ethical approval is not required.

### 2.1 Study eligibility criteria

Studies fulfill the following criteria will be included in this systematic review: randomized controlled trials cohort studies; included patients with locally and regionally advanced or metastatic NSCLC; tested the *EGFR* mutations in blood using any commercial or in-house test; and reported response to EGFR TKI, progression-free survival (PFS), or overall survival (OS).

### 2.2 Literature search and study selection

We will conduct a computerized literature search of PubMed, EMBASE, Cochrane library, and NIHR Health Technology Assessment program from their respective inception to March 2017. The search strategy will consist of the following keywords “non-small cell lung cancer,” “epidermal growth factor receptor,” “plasma,” and “serum.” In addition, we will search the abstracts database of American Society of Clinical Oncology (ASCO) by using the previously mentioned terms. The search strategies are presented in the Supplemental digital content. We will subsequently manually search the bibliographies of included studies and recent narrative reviews for additional studies. There will be no language restrictions. We will consider both published and unpublished studies for inclusion, including those published in abstract form only.

The study selection will be independently carried out by 2 reviewers according to the prespecified criteria. Any discrepancies will be resolved by consensus or by consulting with a 3rd reviewer.

### 2.3 Data extraction

Two reviewers will independently extract data using a predefined data abstraction form with a 3rd reviewer contacted in case of disagreement. The following data will be extracted from each study: study information (ie, title, authors, location, publication date, patient number, and study duration), patient characteristics (ie, age, gender, smoking status, histology, tumor stage, and ethnicity), biomarker-testing methods, intervention (ie, types of EGFR TKIs, standard treatment), outcomes (ie, response to EGFR TKI, PFS, OS, costs, and quality-adjusted life-years [QALYs]), and study methods. In addition, we will construct 2 × 2 tables that contain the number of true positives (patients responding to EGFR TKI and with *EGFR* mutations detected in blood samples), true negatives (patients not responding to EGFR TKI and without *EGFR* mutations detected by blood samples), false positives (patients not responding to EGFR TKI and with *EGFR* mutations detected in blood samples), and false negatives (patients responding to EGFR TKI and without *EGFR* mutations in blood samples). We will consult the authors of original studies if any information mentioned above was not provided in the studied identified.

### 2.4 Quality assessment

The methodological quality of the included studies will be appraised by 2 authors independently, with disagreement resolved by discussion with a 3rd reviewer. The quality of randomized controlled trials will be assessed with the Cochrane Collaboration tool for assessing risk of bias.^[14]^ The quality of cohort studies will be assessed with the Newcastle–Ottawa Scale (NOS).^[15]^

### 2.5 Data analysis for clinical effectiveness

Blood *EGFR* mutation test accuracy for predicting response to TKI treatment will use the response to TKI treatment as reference standard, and calculate sensitivity, specificity, positive likelihood ratio, and negative likelihood ratio for each study according to the above-mentioned 2 × 2 tables. The association between the status of *EGFR* mutations and clinical outcomes of EGFR TKI treatment will be measured by risk ratio for overall response rate, and hazard ratio for PFS and OS. We will pool sensitivity, specificity, positive likelihood ratio, negative likelihood ratio, risk ratio, and hazard ratio using the fixed-effect model unless there is evidence of heterogeneity (*P* ≤ 0.1), in which case random-effects models will be used. Heterogeneity will be explored by the Q-test, with degree of freedom equal to the number of analyzed studies minus 1.^[16]^ A *P* value of 0.10 or below in the Q-test indicates the presence of heterogeneity across studies. We will assess the clinical outcomes of EGFR TKI treatment according to blood *EGFR* mutation testing methods by subgroup analysis. Subgroup differences will be tested using the approach described by Borenstein et al.^[17]^ Tests for subgroup differences will be based on random-effects models which have a lower risk of false-positive results than subgroups assessed in a fixed-effect model.

We will perform sensitivity analyses to assess the robustness of the final results by excluding studies collecting tumor tissue after the initiation of chemotherapy, studies with high risk of bias, and studies with sample-size less than 50. Publication bias will be examined through visual inspection of funnel plot asymmetry if more than 5 studies are involved in the meta-analysis.^[14]^ The funnel plot asymmetry will be evaluated by Egger test.^[18]^ Data analysis for clinical effectiveness will be carried out by STATA Version12.0 (STATA Corporation, College Station, TX) and MetaAnalyst Version Beta 3.13 (Tufts Medical Centre, Boston, MA), with a 2-tailed significance level of 0.05, except for the assessment of heterogeneity (α = 0.10).

## 3 Discussion

Molecular biomarkers for predicting disease risk,^[19,20]^ prognosis and treatment efficacy,^[21]^ comparative effectiveness of different treatments,^[22]^ and cost-effectiveness of treatment strategies^[23]^ play an increasingly important role in the era of precision medicine. This systematic review focuses on *EGFR* mutation testing methods in NSCLC treatment. Our previous study indicated that blood, in particular serum, is a good substitute when tumor tissue is absent or insufficient for testing *EGFR* mutations to guide EGFR TKIs treatment in patients with NSCLC.^[24]^ However, this study neither evaluated the accuracy of different *EGFR* mutation testing methods in blood samples for predicting response to EGFR TKIs nor performed subgroup analysis for the associations of *EGFR* mutations with clinical outcomes according to blood *EGFR* mutation testing methods. Therefore, we plan to undertake the present study to make up the gap in current research. This study will determine the best blood *EGFR* mutation testing method to help clinicians to select NSCLC patients who are most likely to benefit from EGFR TKIs treatment when tumor tissue is absent or insufficient.

## Supplementary Material

Supplemental Digital Content

## References

[1] Torre LA Bray F Siegel RL Global cancer statistics, 2012. CA Cancer J Clin 2015;65:87–108. 25651787 10.3322/caac.21262

[2] Iressa (Gefitinib) Safety and Side Effects Information. Available At: http://www.iressa.com/About-Iressa.Html [Accessed February 11, 2015].

[3] Mitsudomi T Morita S Yatabe Y Gefitinib versus cisplatin plus docetaxel in patients with non-small-cell lung cancer harbouring mutations of the epidermal growth factor receptor (Wjtog3405): an open label, randomised phase 3 trial. Lancet Oncol 2010;11:121–8. 20022809 10.1016/S1470-2045(09)70364-X

[4] Shepherd FA Pereira JR Ciuleanu T Erlotinib in previously treated non-small-cell lung cancer. N Engl J Med 2005;353:123–32. 16014882 10.1056/NEJMoa050753

[5] Costa DB Kobayashi S Tenen DG Pooled analysis of the prospective trials of gefitinib monotherapy for eGFR-mutant non-small cell lung cancers. Lung Cancer 2007;58:95–103. 17610986 10.1016/j.lungcan.2007.05.017 PMC2551312

[6] Bai H Mao L Wang HS Epidermal growth factor receptor mutations in plasma DNA samples predict tumor response in Chinese patients with stages IIIB to IV non-small-cell lung cancer. J Clin Oncol 2009;27:2653–9. 19414683 10.1200/JCO.2008.17.3930

[7] Yung TK Chan KC Mok TS Single-molecule detection of epidermal growth factor receptor mutations in plasma by microfluidics digital PCR in non-small cell lung cancer patients. Clin Cancer Res 2009;15:2076–84. 19276259 10.1158/1078-0432.CCR-08-2622

[8] Kimura H Kasahara K Shibata K eGFR mutation of tumor and serum in gefitinib-treated patients with chemotherapy-naive non-small cell lung cancer. J Thorac Oncol 2006;1:260–7. 17409866 10.1016/s1556-0864(15)31577-x

[9] Kimura H Suminoe M Kasahara K Evaluation of epidermal growth factor receptor mutation status in serum DNA as a predictor of response to gefitinib (Iressa). Br J Cancer 2007;97:778–84. 17848912 10.1038/sj.bjc.6603949 PMC2360394

[10] Xu F Wu J Xue C Comparison of different methods for detecting epidermal growth factor receptor mutations in peripheral blood and tumor tissue of non-small cell lung cancer as a predictor of response to gefitinib. Onco Targets Ther 2012;5:439–47. 23251095 10.2147/OTT.S37289 PMC3525047

[11] Kuang Y Rogers A Yeap BY Noninvasive detection of eGFR T790m in gefitinib or erlotinib resistant non-small cell lung cancer. Clin Cancer Res 2009;15:2630–6. 19351754 10.1158/1078-0432.CCR-08-2592 PMC2727796

[12] Shamseer L Moher D Clarke M Preferred reporting items for systematic review and meta-analysis protocols (PRISMA-P) 2015: elaboration and explanation. BMJ 2015;349:g7647. 25555855 10.1136/bmj.g7647

[13] Mao C Yuan J Zhang Y eGFR Mutation Testing in Blood for Guiding eGFR Tyrosine Kinase Inhibitor Treatment in Patients with Non-Small Cell Lung Cancer: A Systematic Review and Meta-Analysis. Prospero 2017:Crd42017055263. Available from http://www.crd.york.ac.uk/Prospero_Rebranding/Display_Record.Asp?Id=Crd42017055263 [Accessed at 16 January 2017]. 10.1097/MD.0000000000006151 PMC5319537 28207548

[14] Higgins JPT Green S Cochrane Handbook for Systematic Reviews of Interventions Version 5.1.0 [updated March 2011]. The Cochrane Collaboration, 2011 Available from www.handbook.cochrane.org [Accessed at 1 June 2014].

[15] Wells G Shea B O’Connell D The Newcastle-Ottawa Scale (Nos) for Assessing the Quality of Nonrandomised Studies in Meta-Analyses. Available at: http://www.ohri.ca/Programs/Clinical_Epidemiology/Oxford.Asp [Accessed 1 June 2014].

[16] CWG The combination of estimates from different experiments. Biometrics 1954;10:101–29.

[17] Borenstein M Hedges LV Higgins JPT Introduction to Meta-Analysis. Chichester (UK): John Wiley & Sons; 2008.

[18] Egger M Davey Smith G Schneider M Bias in meta-analysis detected by a simple, graphical test. BMJ 1997;315:629–34. 9310563 10.1136/bmj.315.7109.629 PMC2127453

[19] Zhang HF Qiu LX Chen Y ATG16L1 T300A polymorphism and Crohn's disease susceptibility: evidence from 13,022 cases and 17,532 controls. Human Genetics 2009;125:627–31. 19337756 10.1007/s00439-009-0660-7

[20] Mao C Qiu LX Zhan P MnSOD Val16Ala polymorphism and prostate cancer susceptibility: a meta-analysis involving 8,962 subjects. J Cancer Res Clin Oncol 2010;136:975–9. 20012093 10.1007/s00432-009-0742-x PMC11828318

[21] Mao C Liao RY Chen Q Loss of PTEN expression predicts resistance to EGFR-targeted monoclonal antibodies in patients with metastatic colorectal cancer. Br J Cancer 2010;102:940. 20160728 10.1038/sj.bjc.6605575 PMC2833261

[22] Li BZ Threapleton DE Wang JY Comparative effectiveness and tolerance of treatments for Helicobacter pylori: systematic review and network meta-analysis. BMJ 2015;351:h4052. 26290044 10.1136/bmj.h4052 PMC4541168

[23] Westwood M Joore M Whiting P Epidermal growth factor receptor tyrosine kinase (EGFR-TK) mutation testing in adults with locally advanced or metastatic non-small cell lung cancer: a systematic review and cost-effectiveness analysis. Health Technol Assess 2014;18:1–66. 10.3310/hta18320 PMC4781337 24827857

[24] Mao C Yuan JQ Yang ZY Blood as a Substitute for Tumor Tissue in Detecting EGFR Mutations for Guiding EGFR TKIs Treatment of Nonsmall Cell Lung Cancer A Systematic Review and Meta-Analysis. Medicine 2015;94:e775. 26020382 10.1097/MD.0000000000000775 PMC4616411

