# Supplementary material for: EGFR mutation testing in blood for guiding EGFR tyrosine kinase inhibitor treatment in patients with nonsmall cell lung cancer: A protocol for systematic review and meta-analysis
Source: Medicine (Baltimore). 2017 Feb 17;96(7):e6151. doi: 10.1097/MD.0000000000006151 (PMC5319537; doi:10.1097/MD.0000000000006151)
Supplement: Supplemental Digital Content [file medi-96-e6151-s001.pdf]

Supplemental digital content

Search strategies for primary databases

Medline strategy (ovid)

1. (Mutation or mutated or mutat$).sh.
2. exp mutation/
3. mutation.mp.
4. exp Carcinoma, Non-Small-Cell Lung/
5. Carcinoma, Non-Small-Cell Lung.sh.
6. exp Lung Neoplasms/
7. exp Non Small Cell Lung Cancer/
8. Non Small Cell Lung Cancer.mp. or lung non small cell cancer/
9. epidermal growth factor receptor.mp.
10. exp epidermal growth factor receptor/
11. EGFR.mp.
12. serum.mp. or Serum.tw. or Serum.sh.
13. plasma.mp. or Plasma.tw. or Plasma.sh.
14. blood.mp. or blood.tw. or blood.sh.
15. Circulat$.tw. or circulating DNA.tw.
16. 1 or 2 or 3
17. 4 or 5 or 6 or 7 or 8
18. 9 or 10 or 11
19. 12 or 13 or 14 or 15
20. 16 and 17 and 18 and 19
21. limit 20 to (human)

EMBASE strategy (ovid)

1. (Mutation or mutated or mutat$).mp.
2. exp mutation/
3. exp Carcinoma, Non-Small-Cell Lung/
4. exp Lung Neoplasms/
5. exp Non Small Cell Lung Cancer/
6. Non Small Cell Lung Cancer.mp. or lung non small cell cancer/
7. epidermal growth factor receptor.mp. or exp epidermal growth factor receptor/
8. EGFR.mp.
9. Serum.mp. or Serum.tw.
10. Plasma.mp. or Plasma.tw.
11. Blood.mp. or blood.tw.
12. Circulat$.tw. or circulating DNA.tw.
13. 1 or 2
14. 3 or 4 or 5 or 6
15. 7 or 8
16. 9 or 10 or 11 or 12
17. 13 and 14 and 15 and 16

18. limit 17 to (human)

The Cochrane library

1. MeSH descriptor: [Mutation] explode all trees
2. mutated or mutat$.:ti,ab,kw
3. MeSH descriptor: [Carcinoma, Non-Small-Cell Lung] explode all trees
4. MeSH descriptor: [Lung Neoplasms]explode all trees
5. MeSH descriptor: [Non Small Cell Lung Cancer] explode all trees
6. (Non Small Cell Lung Cancer or lung non small cell cancer):ti,ab,kw
7. (epidermal growth factor receptor) :ti,ab,kw
8. MeSH descriptor: [epidermal growth factor receptor] explode all trees
9. EGFR. :ti,ab,kw
10. Serum: ti,ab,kw
11. Plasma: ti,ab,kw w.
12. Blood :ti,ab,kw
13. Circulat$.:ti,ab,kw
14. 1 or 2
15. 3 or 4 or 5 or 6
16. 7 or 8 or 9
17. 10 or 11 or 12 or 13
18. 14 and 15 and 16 and 17

醫療衞生研究基金
Health and Medical Research Fund

Research Fund Secretariat, Research Office, Food and Health Bureau
9th Floor, Rumsey Street Multi-storey Carpark Building, 2 Rumsey Street, Sheung Wan, Hong Kong
Tel: (852) 3150 8987 Fax: (852) 2102 2444 E-mail:rfs@fhb.gov.hk

RESEARCH COUNCIL

Chairman

Secretary for Food and Health / Permanent Secretary for Food and Health (Health)
食物及衞生局局長／食物及衞生局常任秘書長(衞生)

Members

Prof Francis CHAN 陳家亮
Prof Annie CHEUNG 張雅賢
Prof FOK Tai-fai 霍泰輝
Prof David HUI 許樹昌
Prof Mary IP 葉秀文
Prof Nancy IP 葉玉如
Prof LAU Yu-lung 劉宇隆
Prof Diana LEE 李子芬
Prof Gabriel LEUNG 梁卓偉
Prof Dennis LO 盧煜明
Prof Alex MOLASIOTIS 莫禮士
Prof Malik PEIRIS 裴偉士
Prof Paul TAM 譚廣亨
Dr Dominic TSANG 曾艾壯
Prof Maurice YAP 葉健雄
Prof YEOH Eng-kiong 楊永強
Dr YU Wai-cho 余衛祖
Secretary for Innovation and Technology or representative 創新及科技局局長或其代表
Director of Health or representative 衞生署署長或其代表
Chief Executive of Hospital Authority or representative 醫院管理局行政總裁或其代表

Our Ref.: FHB/H/41/117 Tel: 3150 8980
Your Ref.: Fax: 2102 2444

17 November 2016

Prof MAO Chen
Research Assistant Professor
The Jockey Club School of Public Health and Primary Care
The Chinese University of Hong Kong
Room 413, 4/F, Postgraduate Education Centre
Prince of Wales Hospital, 30-32 Ngan Shing Street
Shatin, New Territories, Hong Kong

Dear Prof MAO,

Health and Medical Research Fund
Application for Research Grant (Ref: 14153311)
Application Round: December 2015

Thank you very much for your submission to the 2015 application round of the Health and Medical Research Fund.

After careful consideration, I write to inform that the Research Council considered your application titled "*EGFR mutation testing in blood for guiding EGFR tyrosine kinase inhibitor treatment in patients with non-small cell lung cancer: a systematic review and cost-effectiveness analysis*" as recommended for support subject to clarifications / amendments.

You are required to provide a detailed response to each of the enclosed comments of the Grant Review Board and expert reviewer(s) in a structured and consistent manner. Please note that comments of the Grant Review Board should take precedence. Budget revision other than as specified in the Grant Review Board report will not be considered. Further assessment will then be undertaken before a final decision is made.

Please note that this letter is not a formal approval of funding. You will be informed of the final funding decision in due course. This will be based on the satisfactory response of the applicant to the Grant Review Board and expert referee(s)' comments. The Research Council's funding decision is final.

The Government may require further information to complete the processing of this grant application. If the applicant is unable to provide such information by the deadline stated, or the information is found to be incomplete or inaccurate, the processing of the application may be delayed or the award of funding may be withdrawn.

In particular, you are reminded that copies of all relevant licences, permits, certificates, ethics/regulatory approvals and evidence for accessing third-party data to enable the completion of the project are required. Failure to provide documentary evidence of any of these items in a timely manner may result in the provisional offer of funding being withdrawn.

Please note that the comments enclosed are those of the Grant Review Board as a whole and do not reflect the personal opinions of any individual member.

Please ensure that your response to the comments of Grant Review Board is received by the Secretariat on or before **8 December 2016**. Further revision, unless requested by the Secretariat, after the deadline is not accepted. The above-mentioned regulatory/ethics approvals and/or evidence for accessing third-party data shall be submitted to the Secretariat no later than **8 February 2017**. Incomplete or late reply will not be considered.

Should you have any queries, please contact Dr Pauline LUI Po-yee at 3150 8980 or email to ppylui@fhb.gov.hk.

Yours sincerely,

Dr Edmond MA
Consultant (Research Office)
Food and Health Bureau

c.c. Office of Research and Knowledge Transfer Services, CUHK – Enclosures not attached.
